# Rapidly enlarging port-site mass after thoracoscopic lung resection: desmoid-type fibromatosis mimicking pleural recurrence

**DOI:** 10.1186/s44215-026-00270-5

**Published:** 2026-08-05

**Authors:** Go Kamimura, Masaya Aoki, Tomohiko Matsushita, Satomi Imamura, Shoichiro Morizono, Yuto Nonaka, Aya Takeda, Koki Maeda, Toshiyuki Nagata, Kazuhiro Ueda

**Affiliations:** Department of General Thoracic Surgery, Graduate School of Medical and Dental Sciences Kagoshima University, 8-35-1, Sakuragaoka, Kagoshima City, Kagoshima Pref 892-8520 Japan

**Keywords:** Desmoid-type fibromatosis, Thoracoscopic port site, Chest wall tumor, Lung cancer surgery, Pleural dissemination, Differential diagnosis

## Abstract

**Background:**

Desmoid-type fibromatosis is a rare, benign fibroblastic neoplasm characterized by infiltrative growth and a tendency for local recurrence despite the absence of metastatic potential. It has been associated with prior trauma or surgery, and postoperative lesions arising in the chest wall can present a major diagnostic challenge. In patients after lung cancer resection, a new pleural-based or chest wall mass at a previous port site is often interpreted as pleural dissemination, chest wall recurrence, or port-site implantation, even when the primary tumor is pathologically low risk.

**Case presentation:**

A 70-year-old woman underwent minimally invasive resection for lung adenocarcinoma, and the final pathological stage was pT1miN0M0 (pStage IA1). She remained asymptomatic, and routine postoperative surveillance was performed. At 10 months after surgery, computed tomography revealed a newly developed, well-demarcated soft-tissue mass arising from the chest wall at the thoracoscopic port site, adjacent to the pleura; the mass rapidly enlarged to 3.0 cm by 14 months. Positron emission tomography/computed tomography with fluorine-18 fluorodeoxyglucose showed mild tracer uptake within the lesion (maximum standardized uptake value, 2.5). Although the primary lung adenocarcinoma was pathologically low risk, pleural dissemination or port-site recurrence could not be confidently excluded based on imaging alone. Considering the need for definitive diagnosis and local control, the patient underwent en bloc resection of the lesion with chest wall excision including the seventh rib. Grossly, the tumor was firm and fibrous. Histopathological examination demonstrated a proliferation of bland spindle-shaped cells embedded in abundant collagenous stroma with infiltrative margins, consistent with desmoid-type fibromatosis. The postoperative course was uneventful. As the surgery was performed recently, long-term follow-up data are not yet available.

**Conclusions:**

Desmoid-type fibromatosis can occur at a thoracoscopic port site more than one year after lung cancer surgery and may mimic pleural dissemination or port-site recurrence, even after resection of minimally invasive adenocarcinoma. When malignancy cannot be excluded preoperatively, surgical excision with an adequate margin can establish the diagnosis and achieve local disease control.

## Background

Desmoid-type fibromatosis is a rare fibroblastic proliferation characterized by infiltrative growth and a propensity for local recurrence, despite the absence of metastatic potential [1]. It can arise sporadically and has also been associated with prior trauma or surgical intervention [2, 3]. In thoracic surgery, a newly detected chest wall or pleural-adjacent mass at a previous thoracoscopic port site raises immediate concern for malignant processes, including pleural dissemination, chest wall recurrence, or port-site implantation. This diagnostic uncertainty can be particularly challenging when the primary lung cancer is pathologically low risk, because clinicians must balance the low pretest probability of recurrence against the potentially serious implications of missing an early relapse. We report a case of port-site desmoid-type fibromatosis that radiologically mimicked pleural dissemination after resection of minimally invasive lung adenocarcinoma.

## Case presentation

**Fig. 1:**
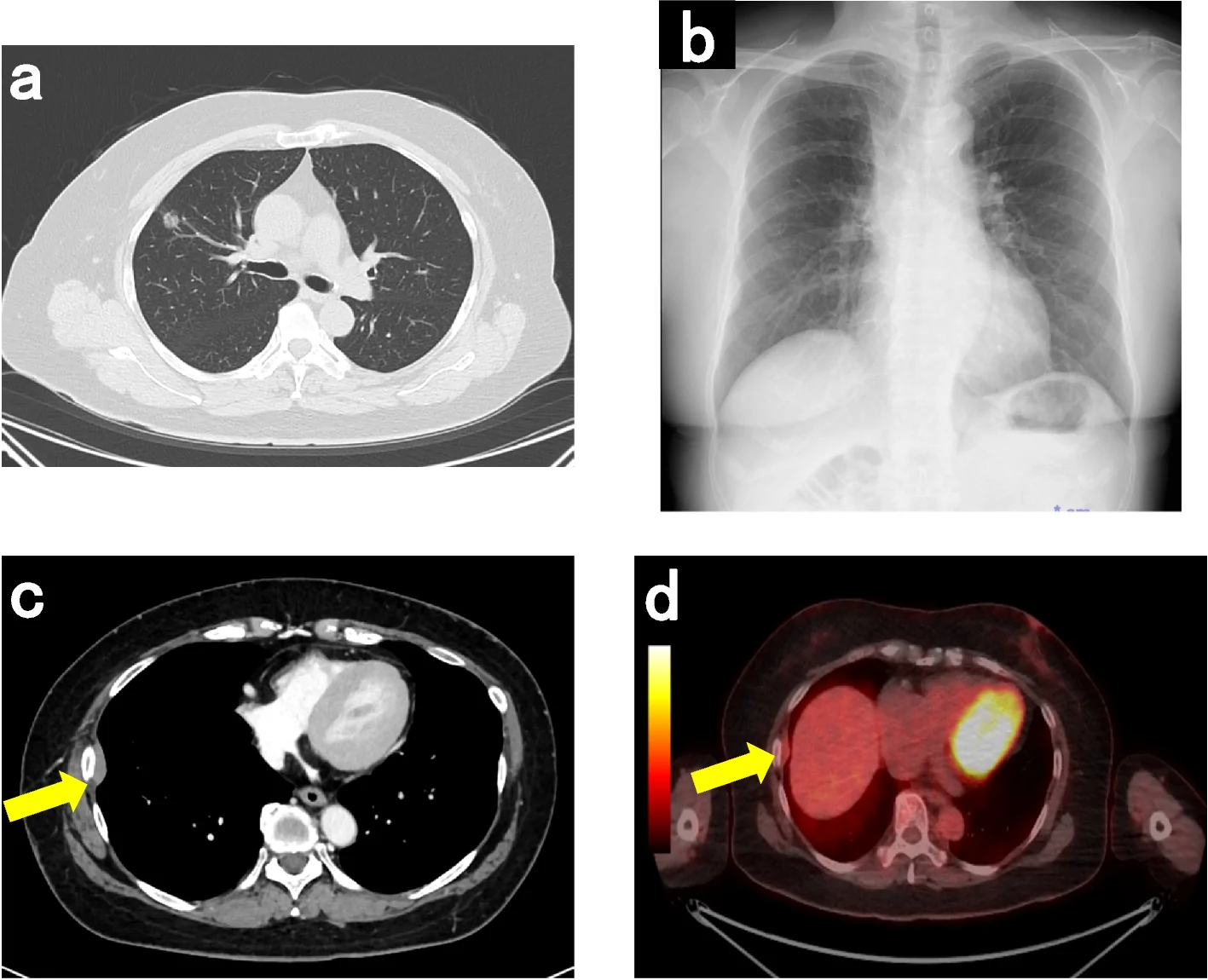
Imaging findings of the primary lung cancer and the postoperative port-site lesion. **a** Preoperative chest computed tomography demonstrating the primary lung adenocarcinoma. **b** Upright chest radiograph demonstrating a chest wall mass abutting the pleura at a previous thoracoscopic port site. **c** Contrast-enhanced computed tomography demonstrating a chest wall mass adjacent to the thoracoscopic port site, abutting the pleura. **d** Positron emission tomography/computed tomography with fluorine-18 fluorodeoxyglucose showing mild uptake in the lesion (maximum standardized uptake value, 2.5)

A 70-year-old woman underwent sublobar resection for lung adenocarcinoma. The initial surgery was performed using a three-port video-assisted thoracoscopic approach with a utility incision up to 4 cm, and two additional port incisions measuring approximately 2 cm.　Preoperative computed tomography showed a small pulmonary lesion (Fig. 1a), and the final pathological diagnosis was minimally invasive adenocarcinoma, pT1miN0M0 (pStage IA1). Her postoperative course was uneventful, and she was followed with routine surveillance imaging.

At 10 months after surgery, computed tomography revealed a newly developed soft-tissue lesion in the chest wall adjacent to a prior thoracoscopic port site, abutting the pleura. The patient was asymptomatic, and there were no laboratory findings suggestive of infection. The lesion enlarged progressively and reached 3.0 cm by 14 months. Upright chest radiography and contrast-enhanced computed tomography confirmed a chest wall mass abutting the pleura at the port site (Fig. 1b, 1c). Positron emission tomography/computed tomography with fluorine-18 fluorodeoxyglucose demonstrated mild uptake (maximum standardized uptake value, 2.5) (Fig. 1d). Although the primary tumor was pathologically low risk, pleural dissemination or port-site recurrence could not be excluded based on imaging alone.

### Surgical approach and intraoperative findings

To minimize potential tumor seeding, a new thoracoscopic port was created at a site distant from the chest wall mass. Thoracoscopic inspection of the pleural cavity was performed, and the chest wall lesion was also visualized from the intrathoracic side. No additional pleural nodules or malignant-appearing pleural effusion were identified. However, because pleural dissemination or port-site recurrence could not be definitively excluded based on the overall findings, a no-touch en bloc resection was planned. A skin incision was made directly over the lesion, and the tumor was excised en bloc with chest wall resection including the seventh rib, without direct manipulation of the mass.

**Fig. 2:**
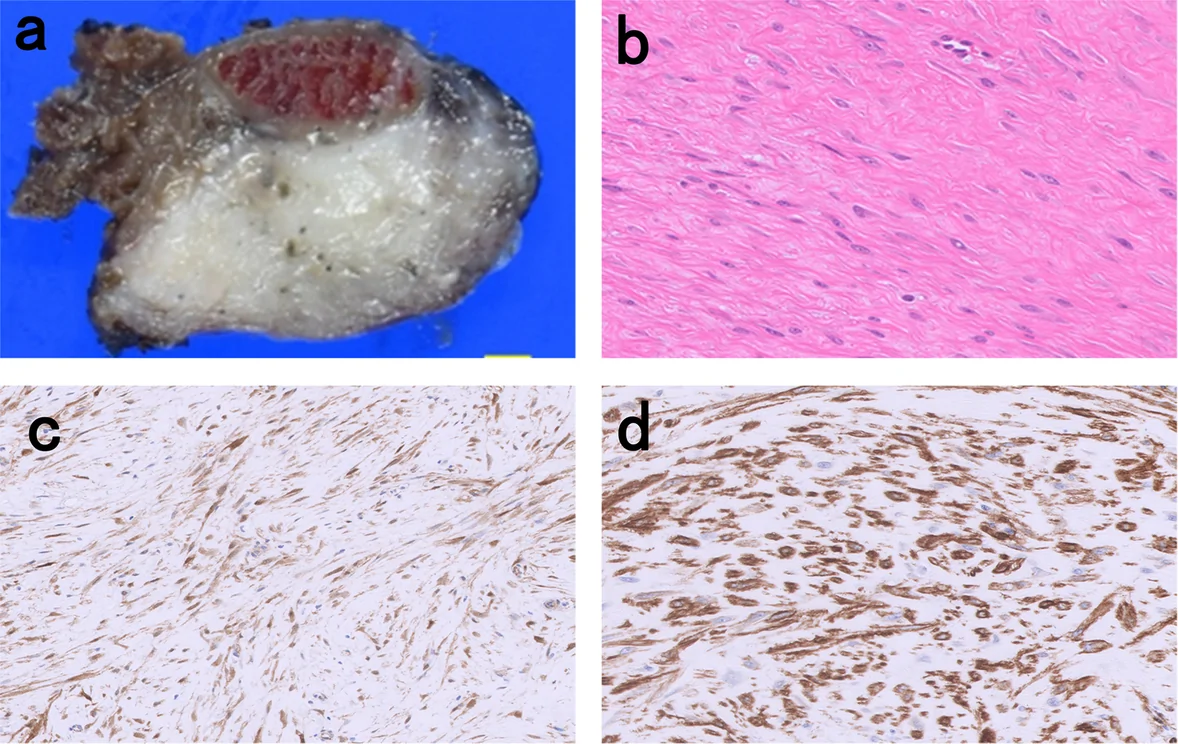
Gross and histopathological findings of the resected tumor. **a** Gross appearance of the en bloc–resected chest wall specimen including the seventh rib. **b** Hematoxylin and eosin staining shows bland spindle-shaped cells arranged in long fascicles within abundant collagenous stroma with an infiltrative growth pattern. **c** Immunohistochemistry demonstrates nuclear β-catenin positivity in tumor cells. **d** Tumor cells are positive for α-smooth muscle actin, consistent with myofibroblastic differentiation

The resected specimen was firm and fibrous (Fig. 2a). Histology showed bland spindle cells in a collagenous stroma with infiltrative growth (Fig. 2b). Immunohistochemistry demonstrated nuclear positivity for β-catenin (Fig. 2c), a characteristic finding supporting desmoid-type fibromatosis, and tumor cells were positive for α-smooth muscle actin, consistent with myofibroblastic differentiation (Fig. 2d). Collectively, these findings were diagnostic of desmoid-type fibromatosis. All surgical resection margins were histologically negative for tumor (R0 resection). The postoperative course was uneventful.

## Discussion

This case highlights a diagnostic pitfall in postoperative surveillance after lung cancer surgery: a growing pleural-adjacent chest wall mass at a thoracoscopic port site may suggest pleural dissemination, chest wall recurrence [4], or port-site implantation, even when the primary tumor is early-stage and pathologically low risk [5]. Although minimally invasive adenocarcinoma (pT1mi) generally carries an excellent prognosis, rare recurrences have been reported even in pT1miN0M0 (stage IA1), including pleural disease diagnosed as metastasis by comprehensive cancer gene panel analysis [6]. Therefore, imaging alone may be insufficient to distinguish benign postoperative processes from malignant recurrence, particularly when interval growth is observed [7].

Desmoid-type fibromatosis is a key non-metastatic but locally aggressive consideration in this setting. It may be triggered by surgical injury and can present as a firm, infiltrative chest wall mass involving adjacent structures. Imaging findings are variable and nonspecific; moreover, mild uptake on positron emission tomography/computed tomography with fluorine-18 fluorodeoxyglucose neither excludes malignancy nor rules out desmoid-type fibromatosis, which may show low-to-moderate uptake [8]. Accordingly, clinical assessment should integrate oncologic background, growth kinetics, anatomic relationship to the pleura/surgical tract, and the feasibility of obtaining a definitive diagnosis.

Management of desmoid-type fibromatosis has shifted toward active surveillance for asymptomatic, stable lesions. However, in postoperative lung cancer patients, observation may be inappropriate when malignancy cannot be excluded and the lesion is enlarging [9]. In our patient, the mass was detected at 10 months and enlarged to 3 cm by 14 months, and its port-site location and pleural adjacency raised concern for dissemination or port-site recurrence. Under these circumstances, en bloc resection provided both definitive diagnosis and local control.

Chest wall excision including a rib is reasonable when adequate margins are required or when the tumor appears inseparable from the chest wall. Although desmoid-type fibromatosis lacks metastatic potential, local recurrence can occur; thus, careful postoperative surveillance and longer follow-up are warranted [10]. Overall, desmoid-type fibromatosis should be included in the differential diagnosis of a new pleural-adjacent chest wall mass at a thoracoscopic port site after lung cancer surgery.

## Conclusion

Desmoid-type fibromatosis can arise from a thoracoscopic port site after lung cancer surgery and may mimic pleural dissemination or port-site recurrence on surveillance imaging. When malignancy cannot be excluded and the lesion enlarges over time, en bloc chest wall resection can establish the diagnosis and achieve local control.

## Data Availability

All data generated or analyzed during this study are included in this published article.

## References

[1] Sbaraglia M, Bellan E, Dei Tos AP. The 2020 WHO classification of soft tissue tumours: news and perspectives. Pathologica. 2020;113:70–84. 10.32074/1591-951X-213. 33179614 PMC8167394

[2] Samejima J, Ito H, Yokose T, Nagashima T. Desmoid-type fibromatosis arising from thoracotomy incision. Clin Case Rep. 2019;8:389–90. 10.1002/ccr3.2634. 32128196 PMC7044401

[3] Shimizu J, Kawaura Y, Tatsuzawa Y, Maeda K, Oda M, Kawashima A. Desmoid tumor of the chest wall following chest surgery: report of a case. Surg Today. 1999;29:945–7. 10.1007/BF02482793. 10489143

[4] Gorospe L, Muñoz-Molina GM, Carvajal-Serrano P, García-Latorre R. Intrathoracic desmoid tumor arising at thoracotomy site mimicking lung cancer pleural recurrence. Ann Thorac Surg. 2017;103:e291. 10.1016/j.athoracsur.2016.10.004. 28219574

[5] Mori T, Yamada T, Ohba Y, Yoshimoto K, Ikeda K, Shiraishi K, et al. A case of desmoid-type fibromatosis arising after thoracotomy for lung cancer with a review of the English and Japanese literature. Ann Thorac Cardiovasc Surg. 2014;20:465–9. 10.5761/atcs.cr.12.02149. 23558226

[6] Nakahashi K, Oizumi H, Suzuki J, Hamada A, Abe K, Sato H. Case report demonstrating the usefulness of a comprehensive cancer panel for the diagnosis of lung adenocarcinoma recurrence that is difficult to differentiate pathologically. J Jpn Assoc Chest Surg. 2019;33:436–41. 10.2995/jacsurg.33.436.

[7] Behera M, Owonikoko TK, Gal AA, Steuer C, Kim S, Pillai RH, et al. Lung adenocarcinoma staging using the 2011 IASLC/ATS/ERS classification: a pooled analysis of adenocarcinoma *in situ* and minimally invasive adenocarcinoma. Clin Lung Cancer. 2011;17:e57–64. 10.1016/j.cllc.2016.03.009. 27137345

[8] Kumar SA, Singh H, Kaman L, Nada R, Mittal BR. Annotating the role of 18F-FDG PET/CT in fibromatoses: a benign masquerader of malignancies—is it really an advantageous tool? Nucl Med Mol Imaging. 2024;58:140–6. 10.1007/s13139-024-00846-5. 38633285 PMC11018563

[9] Desmoid Tumor Working Group. The management of desmoid tumours: a joint global consensus-based guideline approach for adult and paediatric patients. Eur J Cancer. 2020;127:96–107. 10.1016/j.ejca.2019.11.013. 32004793

[10] Gronchi A, Miah AB, Dei Tos AP, Abecassis N, Bajpai J, Bauer S, et al. Soft tissue and visceral sarcomas: ESMO–EURACAN–GENTURIS clinical practice guidelines for diagnosis, treatment and follow-up. Ann Oncol. 2021;32:1348–65. 10.1016/j.annonc.2021.07.006. 34303806

